# Honokiol attenuates lipotoxicity in hepatocytes via activating SIRT3-AMPK mediated lipophagy

**DOI:** 10.1186/s13020-021-00528-w

**Published:** 2021-11-10

**Authors:** Jingxin Liu, Tian Zhang, Jianzhong Zhu, Shuangchen Ruan, Rongsong Li, Bing Guo, Ligen Lin

**Affiliations:** 1 College of Physics and Optoelectronic Engineering, Shenzhen University, Shenzhen, Guangdong China; 2 State Key Laboratory of Quality Research in Chinese Medicine, Institute of Chinese Medical Sciences, University of Macau, Avenida da Universidade, Taipa, Macao, SAR China; 3 College of Health Science and Environmental Engineering, Shenzhen Technology University, 3002 Lantian Road, Pingshan District, Shenzhen, Guangdong China; 4 The Department of Pathophysiology, Guizhou Provincial Key Laboratory of Pathogenesis and Drug Research On Common Chronic Diseases), College of Basic Medical Sciences, Guizhou Medical University, Guiyang, 550025 Guizhou China

**Keywords:** Honokiol, Hepatocytes, Lipid accumulation, Lipophagy, SIRT3, Lipolysis

## Abstract

**Background:**

Non-alcoholic fatty liver disease (NAFLD) is characterized by ectopic accumulation of triglycerides in the liver. Emerging evidence has demonstrated that lipophagy regulates lipid mobilization and energy homeostasis in the liver. Sirtuin 3 (SIRT3), a mitochondrial NAD^+^-dependent deacetylase, modulates the activities of several substrates involving in autophagy and energy metabolism. Honokiol (HK) is a natural lignan from the plants of *Magnolia* genus that exhibits potent liver protective property.

**Methods:**

AML12 was challenged with 500 μM palmitic acid and 250 μM oleic acid mixture solution to induce lipotoxicity. C57BL/6J mice were fed with a choline-deficient high fat diet (CDHFD) to generate liver steatosis. The expression of autophagy-related and AMP-activated protein kinase (AMPK) pathway proteins was evaluated by Western blotting and immunofluorescence staining. Intracellular lipid accumulation was validated by Nile red staining. Molecular docking analysis was performed on AutoDock 4.2.

**Results:**

HK (5 and 10 μM) was found to attenuate lipid accumulation through promoting SIRT3-AMPK-mediated autophagy, mainly on lipid droplets. HK had hydrophobic interaction with amino acid residues (PHE294, GLU323 and VAL324) and NAD^+^. Moreover, HK improved mitochondrial function to enhance lipolysis, through decreasing the acetylated long-chain acyl-CoA dehydrogenase level. In CDHFD-fed mice, HK (2.5 and 10 mg/Kg) treatment obviously prevented lipid accumulation in the liver. And co-treatment of the AMPK inhibitor, Compound C, almost abolished the above changes.

**Conclusions:**

These results suggest that HK could ameliorate lipotoxicity in hepatocytes by activating SIRT3-AMPK-lipophagy axis, which might be a potential therapeutic agent against NAFLD.

## Background

Non-alcoholic fatty liver disease (NAFLD) is prevailing in recent decades, which is closely related to non-alcoholic steatohepatitis (NASH), liver fibrosis and even hepatocellular carcinoma [1]. NAFLD is resulted from the ectopic accumulation of liver lipid, accompanied by lipotoxicity and subsequent metabolic abnormalities [2]. NAFLD occurs when the abnormal accumulation of triglycerides (TG) cannot compensate by the consumption [3]. Autophagy is a cellular recycling process that achieves energy homeostasis through lysosomal dependent degradation. The development of NAFLD is positively associated with impaired autophagy [4, 5]. Lipophagy describes such a process that lipid droplets (LDs) are engulfed into autolysosomes, causing the release of free fatty acids (FFAs) [6]. This actually opens up the possibility to alleviate lipotoxicity in hepatocytes.

Sirtuin 3 (SIRT3), mainly found in mitochondria, is a nicotinamide adenine dinucleotide (NAD^+^)-dependent deacetylase [7]. SIRT3 acts predominantly as a pro-survival factor to protect hepatocytes against oxidative stress [8]. We recently discovered that SIRT3-mediated autophagy promotes lipid mobilization in adipocytes via activating AMP-activated protein kinase (AMPK) [9]. Furthermore, SIRT3 activates lipophagy and chaperon-mediated autophagy to protect hepatocytes from lipotoxicity [10]. These findings imply that SIRT3 regulates lipid homeostasis and is a potential target for NAFLD. Unfortunately, the only ways to activate SIRT3 are calorie limitation and endurance training [11, 12]. The small molecular SIRT3 activators are limited. Honokiol [2-(4-hydroxy-3-prop-2-enyl-phenyl)-4-prop-2-enyl-phenol, HK], a natural lignan ubiquitous in the *Magnolia* genus, is traditional used in Asian ethnic medicines [13, 14]. HK was demonstrated to alleviate hepatic steatosis in various models [15–18]. HK acts as an activator of SIRT3 to reverse cardiac hypertrophy and alleviate oxidative stress [19–21]. Additionally, the binding of HK and SIRT3 activates AMPK to regulate in cellular energy homeostasis [20]. These findings suggest a possible role of HK in regulating hepatic lipid homeostasis.

To validate the hypothesis that HK stimulates lipid mobilization in hepatocytes by promoting SIRT3-mediated lipophagy, we evaluated the regulative effect of HK in lipid challenged hepatocytes and in the liver from choline-deficient high fat diet (CDHFD)-fed mice, and explored the role of SIRT3 in HK induced lipophagy and enhanced mitochondrial function.

## Materials and methods

### Materials

Dulbecco’s Modified Eagle Medium (DMEM), penicillin–streptomycin (P/S), fetal bovine serum (FBS), phosphate-buffered saline (PBS) and 0.25% (w/v) trypsin–EDTA were purchased from Gibco (Gaithersburg, MD, USA). ITS-G (5 mg/mL insulin, 5 mg/L transferrin, 5 μg/L selenious acid) was offered by Peiyuan Biotechnology (Shanghai, China). 3-(4,5-dimethylthiazol-2-yl)-2,5-diphenyltetrazolium bromide (MTT), HK, puromycin, oleic acid, palmitic acid, fatty acid free bovine serum albumin, isoproterenol, DAPI, Oil Red-O, and Free Glycerol Reagent were offered by Sigma–Aldrich (St. Louis, MO, USA). Lipofectamine 3000 Reagent, BCA protein assay kit, SuperSignal West Femto Maximum Sensitivity Substrate and Texas Red-conjugated secondary antibodies were bought from Thermo-Fisher (Grand Island, NY, USA). RIPA lysis buffer and ad-mCherry-GFP-LC3 (#C3011) were offered by Beyotime Biotechnology (Shanghai, China). Triton X-100 and PVDF membranes were supplied by Bio-Rad laboratories (Hercules, CA, USA). The shRNA targeting SIRT3 (mouse, sc-61556), scrambled shRNA (mouse, sc-1080600), and shRNA transfection reagent (mouse, sc-108061) were provided by Santa Cruz Biotechnology (Santa Cruz, CA, USA).

### Cell culture and treatments

AML12 cells, obtained from ATCC (Rockville, MD, USA), were cultured in DMEM (supplemented with 10% FBS and ITS-G) in a humidified incubator (5% CO_2_, 37 °C). Palmitic acid and oleic acid were well dissolved with 75% (v/v) ethanol at 55 °C and diluted to 500 μM and 250 μM with DMEM containing 1% fatty acid free bovine serum albumin (w/v), respectively. To make a mixture solution of palmtic acid and oleic acid (P/O), the two solutions were sterilized with 0.2 μm filter membrane after shaking in an incubator for 2 h.

### Cell viability

The viability of AML12 cells was determined by MTT as previously described [10]. The working solution of HK was prepared immediately before use through diluting the stock solution (10 mM in DMSO) with fresh complete medium.

### Immunoblotting

Protein concentration was quantified with a BCA Protein Assay Kit after lysing the cells with RIPA lysis buffer (containing 1% protease inhibitor cocktail and 1% phenylmethane sulfonylflfluoride). Equal amount of proteins (20‒30 μg) were separated using 5–12% SDS-PAGE and then transferred to PVDF membranes. The membranes were firstly blocked with 5% defatted milk for 2 h at room temperature, followed by overnight incubation of specific primary antibodies at 4 °C and further incubation of secondary antibodies for 1 h at room temperature. SuperSignal West Femto Maximum Sensitivity Substrate kit was used to develop the signals. Visualization of the specific protein bands were achieved on the ChemiDoc MP Imaging System, and the bands were quantitated with Image Lab 5.1 (Bio-Rad laboratories, Hercules, CA, USA).

### RNA transfection and adenovirus infection

Cells were transfected with 2 μg shRNA using Lipofectamine 3000. After 6 h, cells were switched into fresh medium and incubated for 24 h. Then, cells were successively selected with puromycin (2 μg/mL) for 6 days and puromycin (4 μg/mL) for another 6 days. The survived cells were pooled together.

Cells (2 × 10^5^) were seeded in 6-well plates and infected with 10 μL Ad-mCherry-GFP-LC3 (multiplicity of infection = 5) using Lipofectamine 3000. After 24 h, the cells were switched to fresh medium a and incubated for an additional 24 h. Then, cells were pooled together for further investigations.

### Confocal immunofluorescence microscopy

Cells were fixed in formalin (10%), blocked with goat serum (2.5%), and incubated with primary antibodies at 4 ºC overnight. Subsequently, cells were incubated with Texas Red-conjugated secondary antibody at room temperature for 2 h. The nuclei were stained with DAPI. Leica TCS SP8 confocal fluorescence microscope (Leica, Buffalo Grove, IL, USA) was used to capture the images.

### Nile red staining

Nile red staining was conducted as previously reported [22]. Briefly, AML12 hepatocytes were fixed with formaldehyde (10%) and stained with Nile red (1 μg/mL). After incubating for 30 min at 4 °C and washing with PBS, the stained LDs were observed with fluorescence microscopy, and quantitated with flow cytometer with excitation and emission wavelength at 530 and 590 nm, respectively.

### Determination of cellular triglycerides

TG content in cell lysate and the liver tissue was determined by using commercial kits (Nanjing Jiancheng Bioengineering Institute, Nanjing, Jiangsu, China) and normalized by protein concentration.

### Molecular docking analysis

Docking was performed on *AutoDock 4.2*. The crystal structure of the quaternary complex (SIRT3, a substrate, NAD^+^, and the specific agonist amiodarone hydrochloride; PDB ID: 5H4D) [23] was employed as the receptor. The protein was firstly prepared at pH 7.4 with all the water molecules removed and corresponding hydrogen atoms added. The 3D structure of HK was downloaded from the *PubChem* database. Gasteiger charge was calculated and AD4 atom type was assigned, and a 50 Å × 48 Å × 40 Å grid box with 375 Å spacing was placed to include the surface of the catalytic cleft with the assistance of amiodarone hydrochloride. The genetic searching algorithm was chosen for docking calculations, and 50 genetic algorithm runs were performed. Other parameters were set as default. The acquired poses were clustered with a tolerance of 2.0 Å.

### Lipid droplets isolation

LDs were isolated from AML12 hepatocytes as described previously [10]. Briefly, AML12 cells were lysed in hypotonic buffer (50 mM HEPES, 1 mM EDTA and 2 mM MgCl_2_, pH 7.4) supplemented with protease inhibitors after scaping and homogenized with 50 strokes in a Dounce homogenizer. After spinning down at 1,500 g for 5 min, post-nuclear fractions were mixed with equal volume of 1.05 M sucrose in isotonic buffer (50 mM HEPES, 100 mM KCl, 2 mM MgCl_2_) and centrifuged at 100,000 g for 2 h to remove Golgi, rough endoplasmic reticulum, mitochondria, and peroxisomes. The acquired supernatant was adjusted to 1 M sucrose in hypotonic buffer and layered on a sucrose gradient (1 mL of 0.75, 0.5, 0.25, 0.125, and 0 M sucrose solution, respectively). The sucrose gradient tube was centrifuged at 100,000 g for 4 h at 4 °C afterwards, followed by collection of LD fractions from the top which were delipidated with acetone and washed with acetone/ether (1:1, v:v). The pellet was dried under nitrogen and resuspended in protein lysis buffer. The protein concentration of LD fractions was analyzed by BCA Protein Assay kit, and subsequent western blotting was performed.

### Cellular thermal shift assay (CETSA)

Cells were lysed after pretreatment with or without HK (10 μM) for 12 h. The lysates were centrifuged at 12,000 g for 10 min at 4 °C after incubating in ice for 10 min. The protein concentration was determined and adjusted to 3 μg/μL using RIPA lysis buffer. Cell lysates (50 μL) were transferred to new tubes and heated for 3 min at various temperature (50–90 °C) on a thermal cycler. After standing in ice for 10 min, soluble proteins were obtained by centrifugation at 12,000 g for 20 min at 4 °C and analyzed by western blotting [24].

### Mitochondrial membrane potential assay

The fluorescent dye Rhodamine123 was employed to detect the mitochondrial membrane potential. Specifically, AML12 cells were cultured in the presence or absence of HK, and stained with Rhodamine123 (10 μM) for 10 min. Then, cells were washed twice with PBS, trypsinized and collected into a 1.5 mL tube. The change of membrane potential was qualitatively observed on an In Cell Analyzer 2000 (GE Healthcare Life Sciences, Chicago, IL, USA).

### Intracellular reactive oxygen species (ROS) detection

Intracellular ROS levels were detected using DCFH-DA as previously described [25]. Briefly, cells (1 × 10^5^) were seeded into 96-well black multitier plates (clear bottom) and then cultured overnight. The cells were treated with or without HK. After 12 h, the cells were incubated with 2',7'-Dichlorodihydrofluorescein diacetate (DCFH-DA, Sigma-Aldrich, 10 μM) at 37 °C in the dark for 15 min. Fluorescence intensity was analyzed through FACS Calibur flow cytometry (BD, Lake Franklin, NJ, USA).

### Isoproterenol-stimulated lipolysis

The lipolysis activity of AML12 cells was measured as described previously [26]. Cells were incubated with 10 μM isoproterenol (stimulated condition) or DMSO (basal condition) at 37 ºC for 2 h. Subsequently, the medium was collected and heated at 85 °C for 10 min. After centrifuged at 2,000 g for 10 min, Clear supernatant (10 μL) was used to determine the free glycerol content using Free Glycerol Reagent. Lipolysis activity was represented by glycerol concentrations and normalized by protein concentration.

### Immunoprecipitation

Immunoprecipitation was performed as described previously [27]. Briefly, cell lysates (30 µg protein) were mixed with the indicated antibody (2 μg) at 4 °C overnight. Then protein A/G-agarose beads (20 μL) were added to the mixture and incubated on a rotator at 4 °C for 4 h. Immune complexes were washed twice with lysis buffer supplemented with complete mini-protease inhibitor cocktail. Bound proteins were boiled in sample preparation buffer for 5 min and then immunoblotting was conducted.

### Ethic

The procedures and operations involved in the animal experiments were conducted under the Animal Ethical and Welfare Committee of University of Macau (No. ICMS-AEC-2014–06) regulation. The male C57BL/6J mice were maintained in the animal facility of Faculty of Health Science, University of Macau. The mice were fed with normal chow diet (18% protein, 4.5% fat, and 58% carbohydrate, Guangdong Medical Lab Animal Center, Guangzhou, Guangdong, China) and water ad libitum under standard conditions (specific-pathogen-free) with air filtration (22 ± 2 °C, 12-h light/12-h dark).

### Animal experimental procedure

According to the body weight, twenty-eight male mice (6‒8 weeks old) were randomly separated into 6 groups (*n* = 3‒5). The vehicle group of mice (RD) were fed with a regular chow diet and intraperitoneally injected with 10 mL/kg polyethylene glycol 400 (PEG 400, Sigma-Aldrich, St. Louis, MO, USA) solution (PEG 400:0.9% saline, 6:4, *v*/*v*). The remaining five groups of mice were fed with a choline-deficient high fat diet (Trophic Animal Feed High‐Tech Co., Nantong, Jiangsu, China) and intraperitoneally injected with PEG 400 solution (CDHFD), 2.5 mg/Kg HK (HKL, 0.25 mg/mL HK in PEG 400 solution), 10 mg/Kg HK (HKH, 1 mg/mL HK in PEG 400 solution), 5 mg/Kg Compound C (CC, 0.5 mg/mL in PEG 400 solution), and the combination of 5 mg/Kg CC and 10 mg/Kg HK (CC + HK, 0.5 mg/mL CC and 1 mg/mL HK in PEG 400 solution), respectively, once a day for consecutive 4 weeks. Blood samples were collected from tail vein under anesthesia (0.5 L/min inhalation of 3% isoflurane). The mice were euthanized by deeply inhaling carbon dioxide, and the livers were dissected.

### Determination of aspartate transaminase (AST) and alanine transaminase (ALT) levels

The levels of AST and ALT in mouse serum were determined by using commercial assay kits (Nanjing Jiancheng, Nanjing, Jiangsu, China) in accordance with the manufacturer's protocols.

### H&E staining and Oil-red O staining of the liver

After fixation in 4% paraformaldehyde, the liver was embedded in paraffin. 5 μm sections were deparaffinized and rehydrated followed by hematoxylin and eosin (H&E) staining and Oil-red O staining as described previously [28].

### Statistical analysis

All experimental data were expressed as mean ± S.D., and sample size for each experiment corresponds to three biological replicates. Data analysis was finished on GraphPad Prism-6 (GraphPad Software, San Diego, CA, USA), where significant differences between groups were evaluated by one-way analysis of variance (ANOVA) followed by Dunnet’s multiple comparisons test (*p* < 0.05 was considered as significant differences). Where statistical significance is evaluated, variance between groups is confirmed to be similar between comparison groups (control vs. experimental) and the statistical analysis is considered appropriate.

## Results

### Honokiol attenuates lipid accumulation in lipotoxic hepatocytes through promoting autophagy

**Fig. 1:**
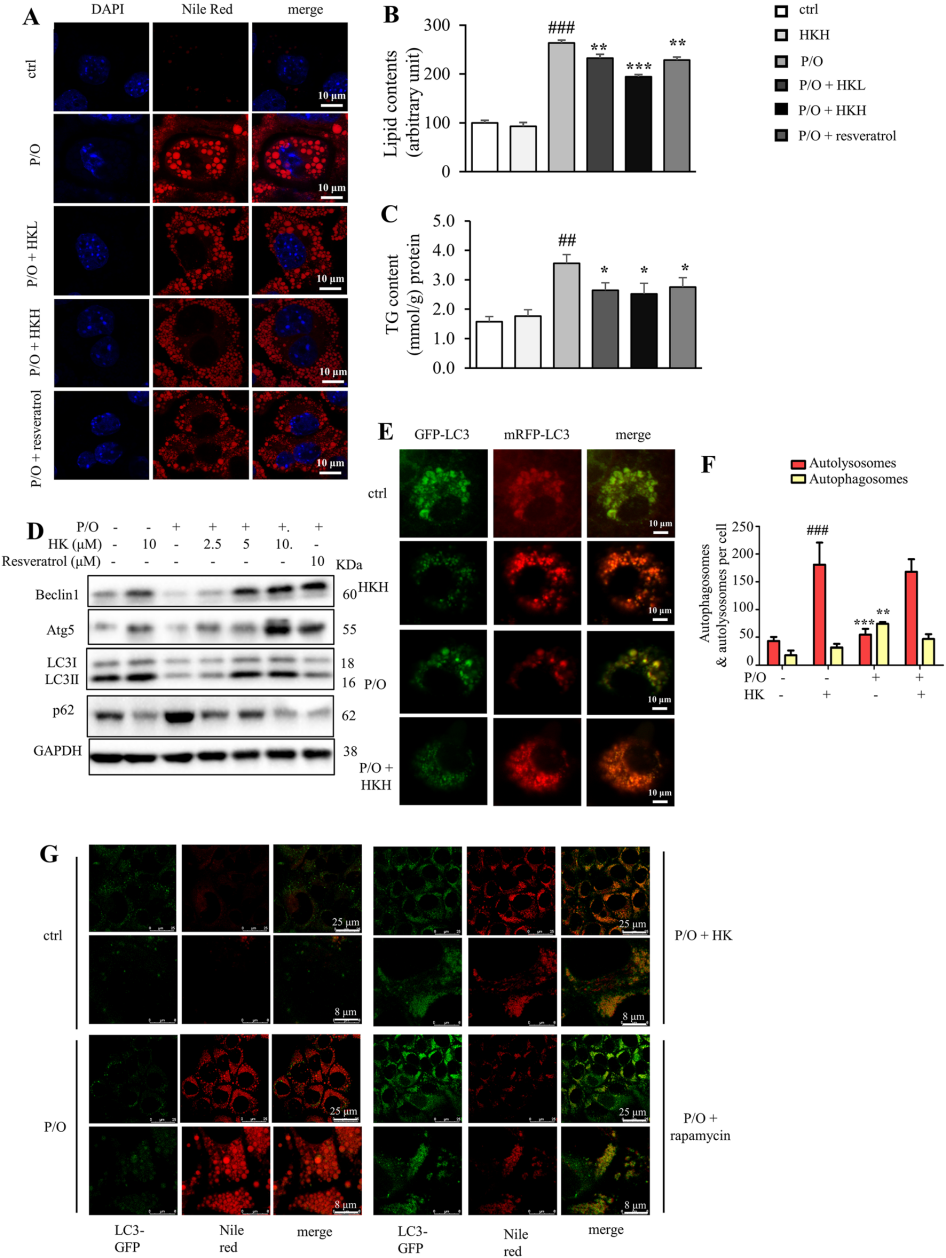
HK attenuates lipid accumulation in lipotoxic hepatocytes through promoting autophagy. **A** Intracellular lipid content visualized with nile red (red) staining. Nuclei were stained with DAPI (blue). Scale bar = 10 μm. Resveratrol (10 μM) was used as a positive control. **B** The lipid content determined by Nile red staining. **C** The cellular TG content. **D** Atg5, Beclin1, LC3 and p62 protein levels in P/O-stimulated AML12 hepatocytes treated without or with various concentrations of HK or 10 µM resveratrol for 24 h. GAPDH was used as a loading control. **E** AML12 cells were infected with the Ad-mCheryy-GFP-LC3, and treated with or without HK. The mRFP-LC3 and GFP-LC3 puncta were visualized on a confocal microscope. Scale bar = 10 μm. **F** The numbers of autophagosomes (yellow puncta) and autolysosomes (red puncta) were quantitated. **G** In P/O-stimulated AML12 hepatocytes, HK treatment induced more LC3-positive puncta (green) on LDs that were visualized with Nile red staining (red). Scale bar = 25 μm. Rapamycin was used as a positive control. Data represented means ± SD. *n* = 6. *p < 0.05, **p < 0.01 and ***p < 0.001, HK or resveratrol vs. P/O treatment. ##*p* < 0.01, ###*p* < 0.001, ctrl vs. P/O treatment. One-way ANOVA was used to calculate the p-values

P/O (palmitic acid and oleic acid mixture) is widely used to induce lipotoxicity in vitro because of more efficient in inducing steatosis and lower cytotoxicity than palmitic acid alone [29, 30]. Herein, lipid accumulation in AML12 cells was induced by stimulation with P/O (the ratio of oleic acid to palmitic acid is 1:2). Firstly, we evaluated the effects of HK against P/O-induced lipid accumulation in AML12 cells. HK did not exhibit obvious cytotoxicity on AML12 cells up to 10 μM [21]. Interestingly, a 2.7-fold increase of lipid content was observed after P/O stimulation, and HK dose-dependently attenuated this effect, which was comparable with the positive control resveratrol (10 µM), as indicated by Nile red staining and its quantitative analyses (Fig. 1A, B). P/O-induced increase of TG content was also reversed by HK treatment (Fig. 1C).

Impaired autophagy results in increased lipid storage in hepatocytes [31]. As shown in Fig. 1D, after P/O-treatment, there is a shrinkage in Beclin1 level and the ratio of LC3-II/LC3-I to approximately 48–75%, whereas p62 level was elevated to 267%, compared with those of the control cells, suggesting impaired autophagy in AML12 cells. Intriguingly, HK treatment reversed the above changes in dose-dependent manners, which was comparable with the positive control resveratrol (Fig. 1D). Meanwhile, HK enhanced autophagy in unstimulated AML12 cells, which was comparable with the positive control resveratrol (Fig. 1D). To check whether HK enhanced autophagic flux, the AML12 cells were infected with mRFP-GFP-LC3 adenovirus to label autophagosomal formation. As shown in Fig. 1E and F, impaired autophagy was reflected by the decreased both red and green puncta in P/O treated AML12 cells. More mRFP-LC3 puncta were observed in HK-treated cells as expected, suggesting that autophagic flux was improved with undisturbed lysosomal function and/or autophagosome-lysosome fusion. Furthermore, the fluorescent images indicated that HK-induced autophagosome formation was largely co-localized with LDs (Fig. 1G). These results indicated that HK mitigates lipid accumulation in lipotoxic hepatocytes through promoting autophagy*.*

### Honokiol attenuates lipid accumulation through SIRT3-mediated autophagy

**Fig. 2:**
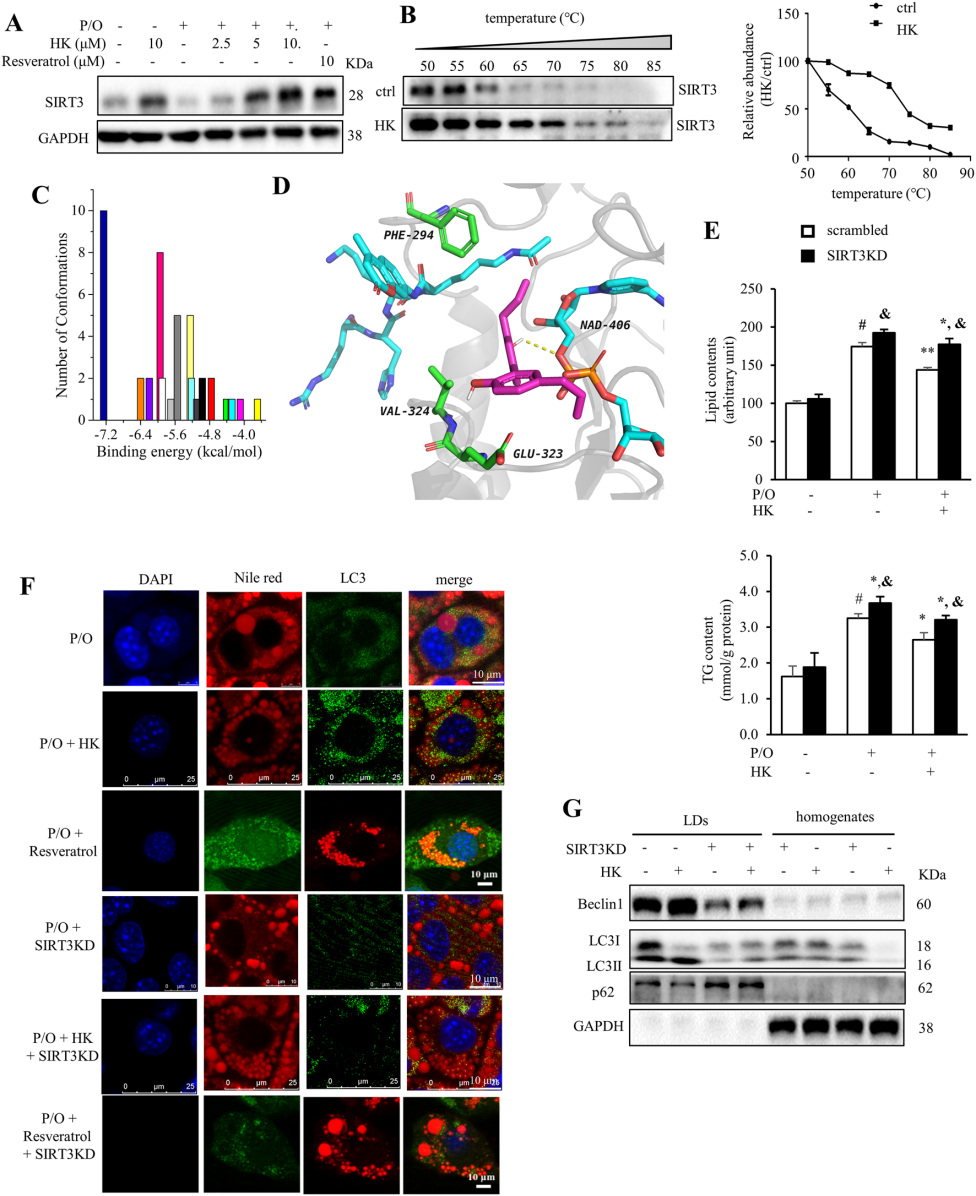
HK attenuates lipid accumulation through SIRT3-mediated autophagy. P/O-stimulated AML12 cells were treated without or with various concentrations of HK for 24 h. **A** SIRT3 protein levels were evaluated. GAPDH was used as a loading control. **B** CETSA was performed on AML12 cells treated with or without HK (10 μM) for 12 h. The SIRT3 protein levels were detected by using Western blotting. Data were normalized to the mean value of the respective group at 50 °C (*n* = 5). **C** Docking analysis of the binding between HK and SIRT3 (PDB ID: 5H4D). Cluster analysis of the docked conformations of HK. A tolerance of 2.0 Å was used. **D** Interactions between HK and residues on SIRT3. The protein was shown in New Cartoon, and small molecules in sticks; the substrate (or NAD^+^), residues, and HK were colored in cyan, green, and magenta, respectively. **E** The scrambled and SIRT3KD cells were treated with or without HK for 24 h. The lipid content was determined by flow cytometry after nile red staining and the cellular TG content were determined by commercial kit. **F** SIRT3 silencing blocked HK treatment-induced co-localization of LC3 puncta (green) on LDs. LDs were visualized with nile red fluorescence. Scale bar = 25 μm. **G** HK treatment activated autophagy mainly on LDs. Data was represented as means ± SD. *p < 0.05 and **p < 0.01, HK vs. P/O treatment. #*p* < 0.05, ctrl vs. P/O treatment. &*p* < 0.05, scrambled vs SIRT3KD groups. One-way ANOVA was used to calculate the p-values

SIRT3 overexpression protects hepatocytes from lipotoxicity though promoting lipophagy and chaperon-mediated autophagy [10]. Interestingly, HK treatment dose-dependently increased SIRT3 level in P/O-treated AML12 cells (Fig. 2A). To experimentally verify the interaction between HK and SIRT3 deacetylase, CETSA was performed on AML12 cells treated with or without HK. Compared to the control cells, the thermal stability of SIRT3 was strongly enhanced by HK at various temperatures (Fig. 2B). To verify the interaction pattern between HK and SIRT3, docking analysis was conducted. Clustering analysis showed the predominant cluster had the lowest binding energy with the best pose owing a − 7.2 kcal/mol (Fig. 2C). HK had hydrophobic interaction with amino acid residues (PHE294, GLU323 and VAL324) and NAD^+^ (Fig. 2D). Additionally, it was hydrogen bonded with an oxygen on the NAD^+^ (Fig. 2D).

To evaluate the role of HK-driven SIRT3 in reducing lipid accumulation in hepatocytes, the SIRT3 knockdown AML12 cell line (SIRT3KD) was generated using shRNA targeting SIRT3. As expected, SIRT3 silencing partially blocked the lipid lowering effects of HK in P/O-stimulated AML12 cells (Fig. 2E). HK treatment increased the expression of LC3II, and reduced lipid content and LD size in P/O-treated AML12 cells; whereas, silencing of SIRT3 almost abrogated the effects of HK, which was comparable with the positive control resveratrol (Fig. 2F).

The LD fraction was isolated from scrambled and SIRT3KD AML12 cells treated with or without HK, and enrichment of LC3-II, Beclin1 and decreased p62 were observed in LDs, but not in homogenates after treatment of HK; and deletion of SIRT3 almost reversed HK-driven activation of autophagy in the isolated LDs (Fig. 2G). These observations indicated HK treatment induced lipophagy rather than bulky autophagy to alleviate lipid accumulation and SIRT3 is required in HK-induced lipophagy.

### Honokiol alleviates lipid accumulation through SIRT3-AMPK-induced autophagy

**Fig. 3:**
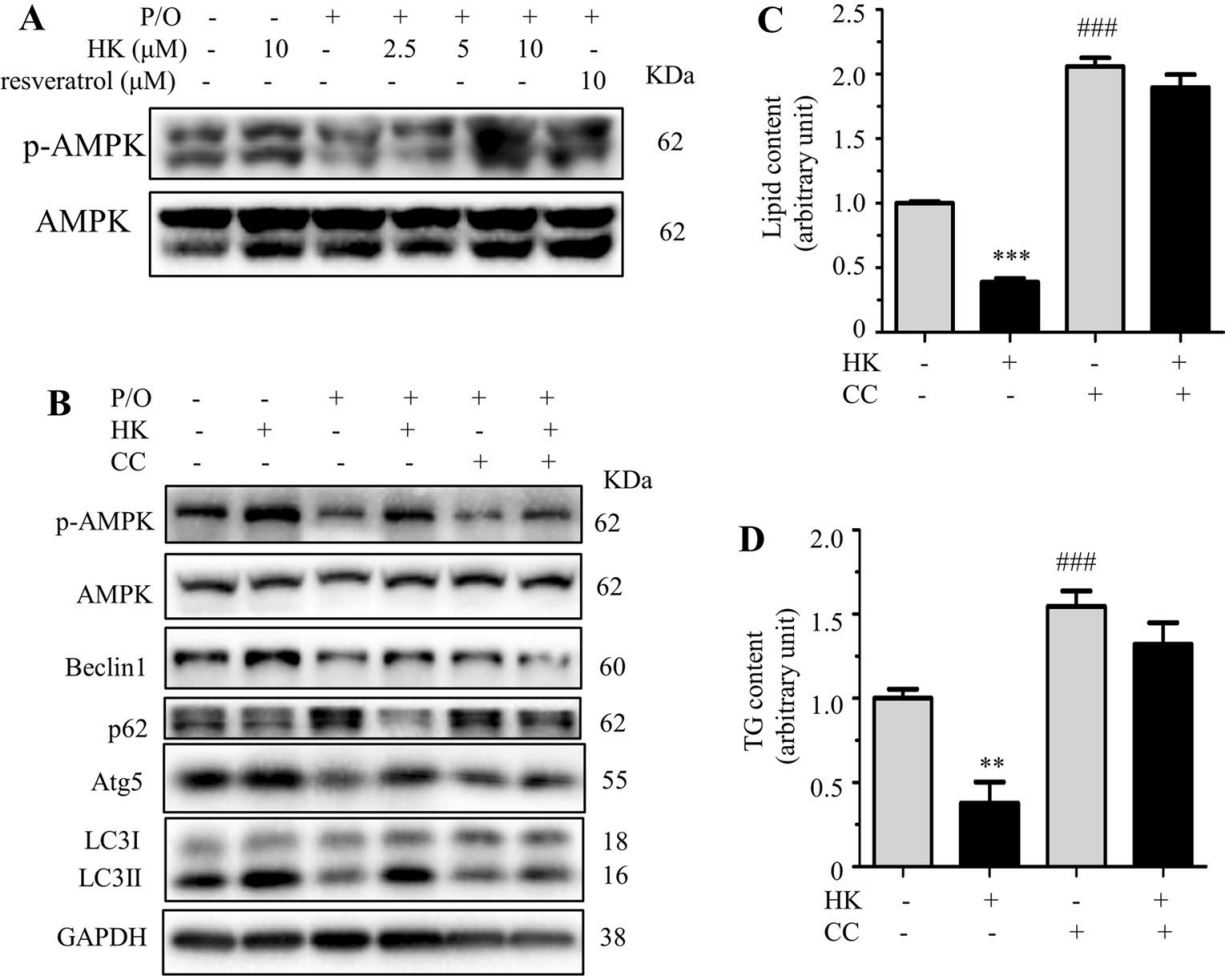
HK alleviates lipid accumulation through SIRT3-AMPK-induced autophagy. P/O-stimulated AML12 cells were treated without or with various concentrations of HK for 24 h. **A** p-AMPK and AMPK protein levels evaluated by Western blotting. GAPDH was used as a loading control. **B** P/O-stimulated AML12 cells were treated without or with HK (10 μM) and CC (10 μM) for 24 h. p-AMPK, AMPK, Beclin1, Atg5, p62 and LC3 protein levels were detected by Western blotting. GAPDH was used as a loading control. **C** The lipid content determined by Nile red staining. **D** The cellular TG content. Data represented means ± SD. **p < 0.01 and ***p < 0.001, HK vs. P/O treatment. ##*p* < 0.001, CC vs. P/O treatment. One-way ANOVA was used to calculate the p-values

SIRT3 activates autophagy through the AMPK pathway in palmitate-stressed hepatocytes [10]. To elucidate the mechanism of HK on SIRT3-mediated autophagy, the phosphorylation level of AMPK was determined in HK treated hepatocytes. HK dose-dependently increased the phosphorylated AMPK level in P/O treated AML12 cells, which was comparable with the positive control resveratrol (Fig. 3A). Compound C (CC), an inhibitor to AMPK, remarkably diminished the effect of HK on activating autophagy (Fig. 3B). As shown in Fig. 3C and D, treatment of CC alone greatly increased lipid and TG contents, and co-treatment of CC and HK reversed the reducing effect of HK on lipid and TG contents, supporting that HK alleviated lipid accumulation in AML12 cells via activating AMPK signaling pathway. These results suggested that the effect of HK on lowering lipid accumulation was mediated through SIRT3-AMPK-mediated autophagy.

### Honokiol attenuates lipid accumulation by restoring mitochondrial function

**Fig. 4:**
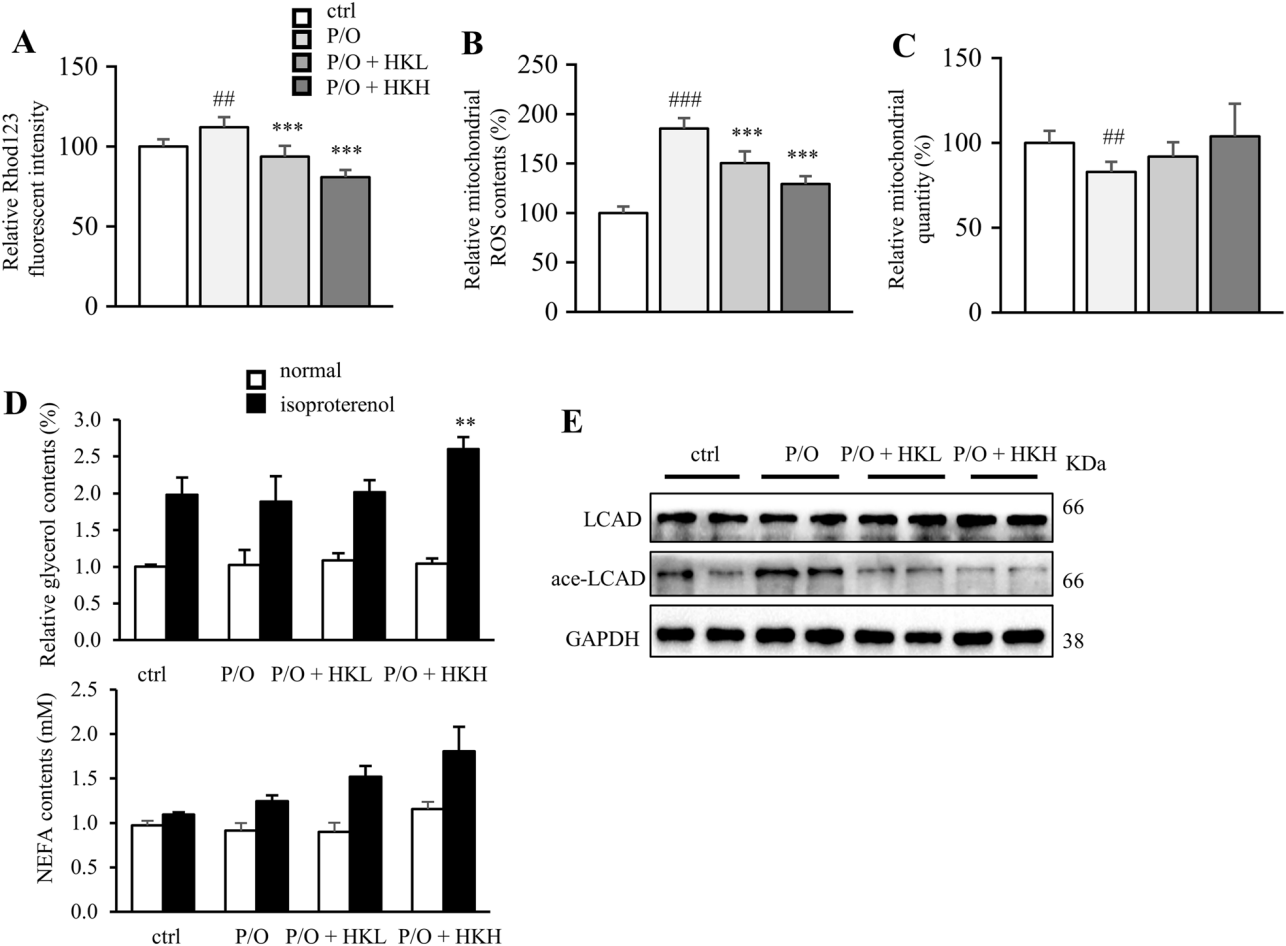
HK attenuates lipid accumulation by restoring mitochondrial function. AML12 cells were treated with or without HK in the presence of P/O for 24 h. **A** The relative Rhod123 fluorescent intensity. **B** Intracellular ROS accumulation levels were determined by DCFH-DA assay. **C** Mitochondria were stained by Mitochondria-green and quantitated by flow cytometry. **D** Relative glycerol content in P/O-stimulated AML12 hepatocytes under both normal and isoproterenol-induced conditions. **E** The acetylated and total LCAD levels. Data was expressed as means ± SD. **p < 0.01 and ***p < 0.001, HK vs. P/O treatment. ##*p* < 0.01 and ###*p* < 0.001 ctrl vs. P/O treatment. One-way ANOVA was used to calculate the p-values

Next, we assumed that the lipid lowering effect of HK was involved in enhanced mitochondrial function. The mitochondrial membrane potential was evaluated by using Rhodamine 123 staining. The results showed that P/O stimulation disrupted mitochondrial membrane potential, whereas HK-treated cells exhibited higher mitochondrial membrane potential, suggesting improved mitochondrial function (Fig. 4A). Meanwhile, lipid challenge led to high level of intracellular ROS and reduced mitochondrial content; whereas, HK treatment alleviated oxidative stress and slightly enhanced mitochondrial biogenesis in P/O-treated AML12 cells (Fig. 4B and C). Activation of autophagy not only shifts lipids to the lysosome for degradation by acid lipases, but enhances lipolysis by neutral lipases [10]. Upon isoproterenol stimulation, hormone-sensitive lipase translocates from the cytosol to the surfaces of intracellular LDs concomitant with the onset of lipolysis, as measured by the release of glycerol and non-esterified fatty acid (NEFA) to the culture medium. Interestingly, high dosage of HK enhanced the glycerol content in isoproterenol-stimulated cells, but not in the control cells (Fig. 4D); while HKH only slightly increased the NEFA level (Fig. 4D). Long-chain acyl-CoA dehydrogenase (LCAD) is a deacetylating substrate of SIRT3 [7]. HK decreased the acetylated LCAD level in a dose-dependently, but did not change the total LCAD level (Fig. 4E). Taken together, HK rescues hepatocytes from P/O-induced lipid accumulation by maintaining mitochondrial function and promoting lipolysis.

### HK alleviated liver steatosis in CDHFD-fed mice

**Fig. 5:**
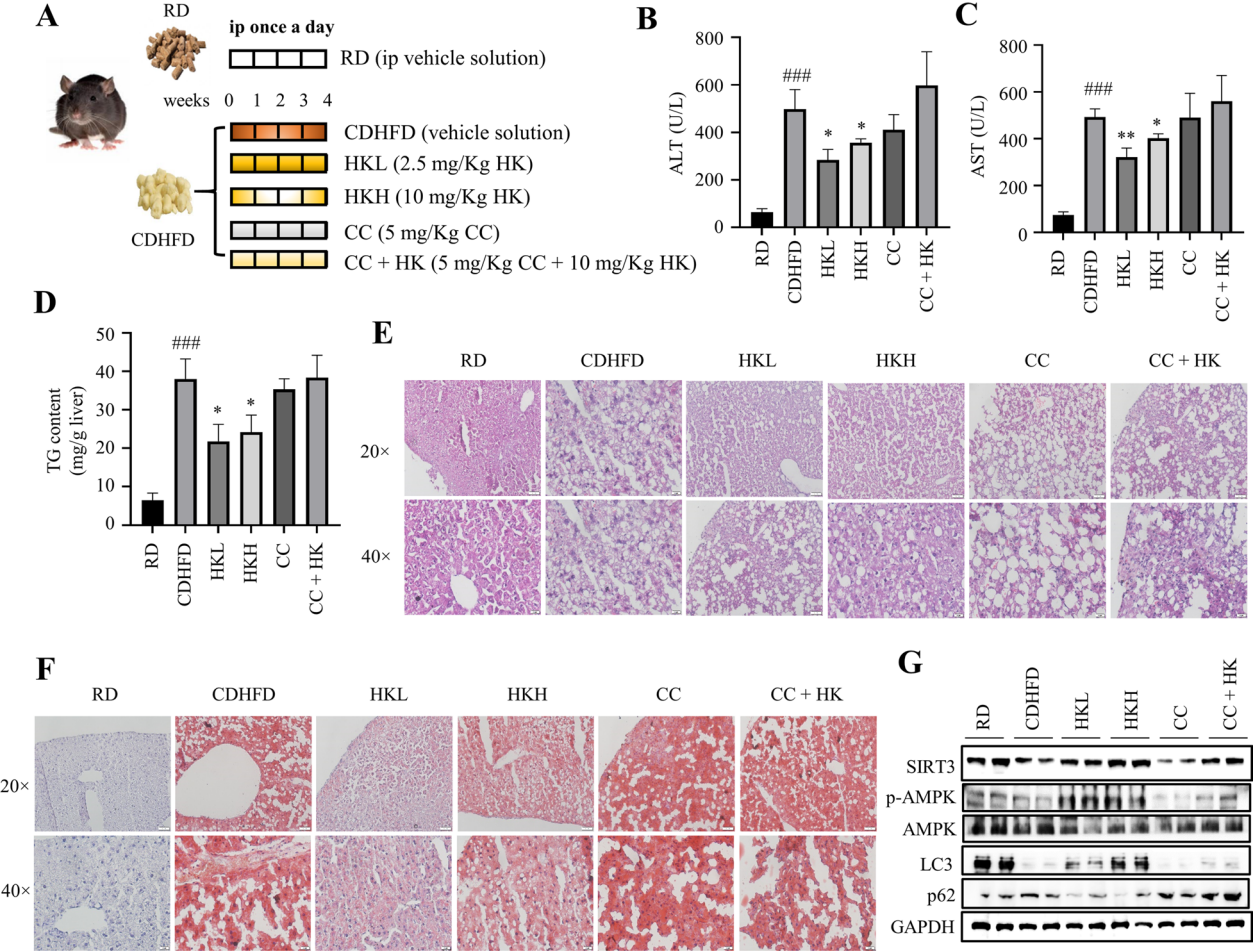
HK alleviate liver steatosis in CDHFD-fed mice. **A** The experimental procedure. HKL: 2.5 mg/Kg HK; HKH: 10 mg/Kg HK; CC: 5 mg/Kg CC; CC + HK: 5 mg/Kg CC and 10 mg/Kg HK. *n* = 3‒5. **B** Serum ALT levels in mice. **C** Serum AST levels in mice. **D** TG content in the liver. **E** Representative images of H&E staining of the liver. Scale bar = 50 µm (top); scale bar = 20 µm (bottom). **F** Representative images of Oil-red O staining of the liver. Scale bar = 50 µm (top); scale bar = 20 µm (bottom). **G** The expression levels of p-AMPK, AMPK, LC3, p62, PLN2, HSC70, LAMP2 and SIRT3 in the liver were determined by Western blots. GADPH was used as a loading control. Data are shown as mean ± SD. *p < 0.05 and **p < 0.01, HK vs. CDHFD. ###*p* < 0.001 RD vs. CDHFD. One-way ANOVA was used to calculate the p-values

To investigate the protective effect of HK against lipotoxicity in vivo, CDHFD-induced liver steatosis mice were recruited (Fig. 5A). Four weeks of CDHFD feeding did not change body weight (data not shown). The serum levels of ALT and AST, as well as the liver TG content, were greatly increased in CDHFD-fed mice when compared with those of RD-fed mice (Fig. 5B‒D), which suggested the liver steatosis model was well generated. Furthermore, the H&E staining and Oil-red O staining of the liver from CDHFD-fed mice exhibited a great increase of lipid content (Fig. 5E and F). As expected, either low or high dosage of HK treatment markedly decreased the serum ALT and AST levels, and the liver TG and lipid contents, compared with those of CDHFD mice (Fig. 5B‒F). These changes were abolished in CC co-treated group (Fig. 5B‒F). The phosphorylation of AMPK and the protein level of SIRT3 were suppressed in the liver from CDHFD mice (Fig. 5G), and HKL and HKH activated AMPK and increased the protein expression of SIRT3 in the liver (Fig. 5G). On the other hand, CDHFD feeding resulted decreased protein expression of LC3 and increased protein expression of p62 in the liver, suggesting impaired autophagy (Fig. 5G). Intriguingly, either low or high dosage of HK treatment activated autophagy in the liver (Fig. 5G). And co-treatment of CC almost reversed the above changes (Fig. 5G). Collectively, these results suggested that HK alleviates liver steatosis in CDHFD-fed mice, which might be mediated through the SIRT3-AMPK axis.

## Discussion

Increasing evidence has uncovered a positive connection between lipophagy and the onset of NAFLD. Organisms regulate free fatty acids release to supply metabolic demand by lipophagy [32]. Impaired autophagy leads to excessive lipid accumulation in the liver to cause hepatic steatosis [33, 34]. HK has been found to activate autophagy in several cancer cells [35–37]. Herein, we found HK attenuates lipid accumulation in lipotoxic hepatocytes through promoting SIRT3-AMPK-induced autophagy and mitochondrial function. Furthermore, the role of HK in treating NAFLD was verified in a CDHFD-induced fatty liver mice model.

Autophagy accounts for a major part of lipolysis in liver. Consequently, autophagy blockage through knockdown of the key autophagic genes like Atgs in hepatocytes, led to an increase of LDs in cells even under normal nutritional conditions [38]. Interestingly, impaired autophagy further deteriorated LDs accumulation, leading to hepatotoxicity and severe steatosis [39]. In our current study, HK was found to activate lipophagy, stimulate lipolysis under isoproterenol treated condition and decrease the acetylated LCAD level. These results indicated that HK protects hepatocytes against lipotoxicity through enhancing lipophagy and lipolysis.

Liver contains a large number of mitochondria, which are the predominant source of intracellular ROS. Excessive ROS accumulation results in cell death through the oxidation of polyunsaturated fatty acids [40]. Mitochondrial homeostasis was interrupted when the hepatocytes were exposed to P/O stimulation, accompanied with decreased mitochondrial content and increased ROS production. Interestingly, high dose of HK maintained mitochondrial integrity in hepatocytes under lipotoxic stress. In fact, mitochondrial biogenesis acts as a critical factor for mitochondrial quantity, and HK was found to facilitate the process by targeting peroxisome proliferator-activated receptor-γ coactivator-1α [41]. Herein, HK did not affect mitochondrial biogenesis, but enhanced mitochondrial function in lipotoxic hepatocytes. SIRT3 may also be involved in mitochondrial renewal and hepatocyte proliferation through mitophagy mechanisms [42]. Future studies are required to fully elucidate how HK regulates mitophagy in the context of hepatocellular lipotoxicity.

In response to a variety of conditions that deplete cellular energy levels, AMPK is activated to coordinate metabolic pathways and balance nutrient supply with energy demand [43]. Previous studies indicated that AMPK activation attenuates hepatic steatosis through suppressing de novo lipogenesis in hepatocytes, increasing fatty acid oxidation in the liver, and promoting mitochondrial function and integrity in adipose tissue [44]. Ablation of AMPK activity in adipose tissue causes a decrease in adipose tissue insulin sensitivity and an increase in liver lipid accumulation [45]. CC is widely used as a cell-permeable ATP-competitive inhibitor of AMPK to revert the positive effects of 5-aminoimidazole-4-carboxamide-1-β-D-ribofuranoside and metformin [46]. CC treatment was reported to increase lipid and TG contents in hepatocytes [47], which is consistent with the current observation. On the contrary, CC has been demonstrated to be with AMPK-independent pharmacological actions. In HFD-fed mice, CC administration was found to reduce hepatic steatosis and ballooning by impairing NOD-, LRR- and pyrin domain-containing protein 3 inflammasome activation and the related inflammation [48].

The most widespread and prevailing model describing the development of NAFLD is the “multiple-hit hypothesis”, where the “first hit”, hepatic lipid accumulation, induces lipotoxicity or steatosis, rendering liver more vulnerable to “subsequent hits” injury, such as mitochondrial dysfunction and oxidative stress, which in turn leads to steatohepatitis and cirrhosis, and eventually hepatocellular carcinoma [49]. In the previous study, we reported that HK scavenges excessive ROS and repairs cellular damages in oxidative injured hepatocytes. Herein, we further found that HK activates lipophagy to promote LD breakdown, leading to reduced lipotoxicity in hepatocytes. Taken together, HK might protect hepatocytes against oxidative stress and alleviate lipotoxicity, rendering it a potential candidate to treat NAFLD against multiple hits.

## Conclusions

**Fig. 6:**
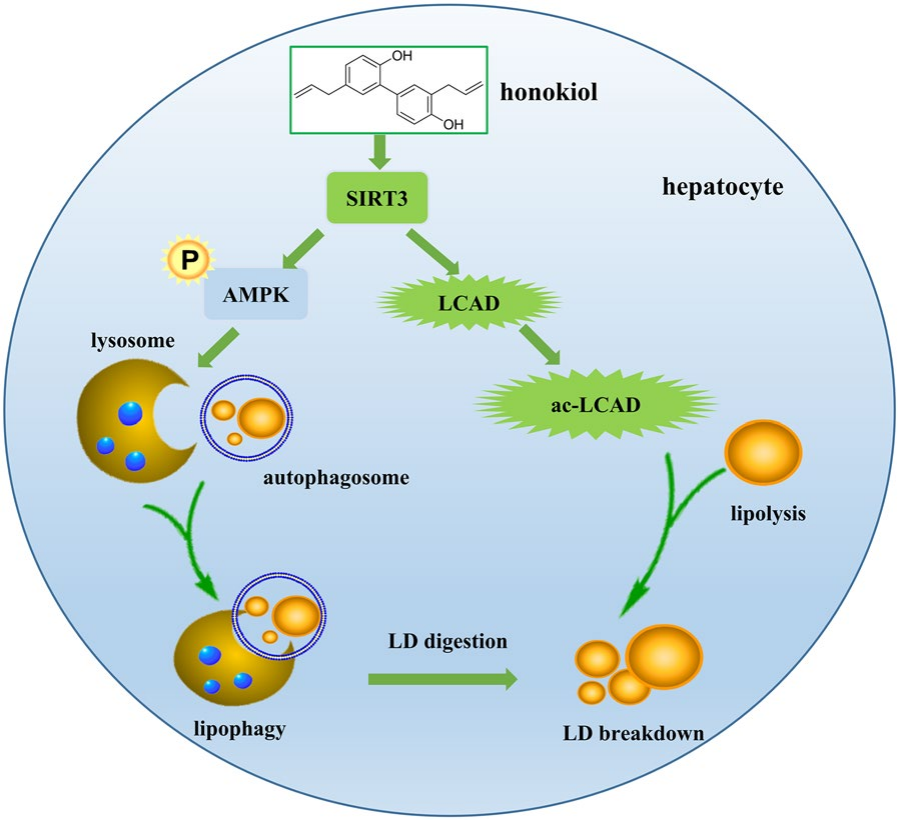
Schematic of the role of HK in protecting hepatocytes against lipotoxicity. HK activates SIRT3, which in turn activates AMPK to enhance autophagy on LDs and deacetylates LCAD to increase fatty acid oxidation in mitochondria, resulting in attenuation of lipotoxicity in hepatocytes

In conclusion, we verified HK protects hepatocytes against lipotoxic stress through enhancing SIRT3-AMPK-induced lipophagy, and maintain mitochondrial morphology and integrity (Fig. 6). HK could be a potential candidate in the treatment of NAFLD.

## Data Availability

Not applicable.

## Abbreviations

ALT: Aminoalanine transaminase
AMPK: AMP-activated protein kinase
AST: Aspartate transaminase
CC: Compound C
CDHFD: Choline-deficient high fat diet
CETSA: Cellular thermal shift assay
FFAs: Free fatty acids
HCC: Hepatocellular carcinoma
HK: Honokiol
LCAD: Long-chain acyl-CoA dehydrogenase
LD: Lipid droplet
3-MA: 3-Methyladenine
NASH: Non-alcoholic steatohepatitis
NAFLD: Non-alcoholic fatty liver disease
P/O: Palmitic acid/oleic acid
ROS: Reactive oxygen species
SIRT3: Sirtuin 3
TG: Triglyceride
